# Measuring Maximum Head Circumference Within the Picture Archiving and Communication System: A Fully Automatic Approach

**DOI:** 10.3389/fped.2021.608122

**Published:** 2021-07-19

**Authors:** Fernando Yepes-Calderon, Frisca Wihardja, Andrea Sloan, Janet Kim, Marvin D. Nelson, J. Gordon McComb

**Affiliations:** ^1^Strategic Business Platforms LLC, Fort Pierce, FL, United States; ^2^Children's Hospital of Los Angeles, Los Angeles, CA, United States; ^3^Division of Neurosurgery, Children Hospital Los Angeles, Los Angeles, CA, United States; ^4^Department of Neurological Surgery and Radiology, Keck School of Medicine, Los Angeles, CA, United States; ^5^Department of Radiology, Children's Hospital Los Angeles, Los Angeles, CA, United States

**Keywords:** medical imaging, automatic diagnosis, maximum head circumference, PACS, clinical imaging methods

## Abstract

This study describes an automatic technique to accurately determine the maximum head circumference (MHC) measurement from MRI studies within the Picture Archiving and Communications System, and can automatically add this measurement to the final radiology report. Participants were selected through a retrospective chart review of patients referred to the neurosurgery clinic. Forty-nine pediatric patients with ages ranging from 5 months to 11 years were included in the study. We created 14 printed ring structures to mirror the head circumference values at various ages along the x-axis of the Nellhaus chart. The 3D-printed structures were used to create MRI phantoms. Analytical obtainment of circumference values from the 3D objects and phantom images allowed for a fair estimation and correction of errors on the image-based-measuring instrument. Then, standard manual MHC measurements were performed and compared to values obtained from the patients' MRI T1 images using the tuned instrument proposed in this document. A *T*-test revealed no statistical difference between the manual assessments and the ones obtained by the automation *p* = 0.357, α = 0.05. This automatic application augments the more error-prone manual MHC measurement, and can add a numerical value to the final radiology report as a standard application.

## 1. Introduction

In addition to length and weight, maximum head circumference (MHC) is a standard measurement obtained in pediatric patients, especially within the first 2 years of life (1, 2).

The MHC provides an indirect idea about health, development, nutrition, and response to treatment (3) Despite the MHC approach's simplicity, it yields important information when accurately estimated and analyzed together with height and weight. The abnormalities that are more often determined by the MHC are hydrocephalus (4), craniosynostosis (5), and microcephaly (6, 7). Nevertheless, more complex associations of skull uncontrolled growth and abnormalities have been reported. That is the case of the HOXA1 gene, and a statistically proven 5% increase in the head's circumference in children with autism (2) with respect to ethnically and aged-matched children. Also, a systematic review of autism spectrum disorder patients revealed that head circumference is bigger in autistic patients than in control individuals; moreover, the authors demonstrated that larger heads are associated with low functioning individuals (8). MHC has also been used to anticipate the degree of intellectual disability in microcephaly patients (6), and a low reading of the head circumference is a risk factor for brain cancer (9).

The manual obtainment of the MHC is an inexact procedure influenced by the degree of patient cooperativeness and the clinicians' thoroughness when attempting to determine the most extended perimeter's correct location. As a result, the intra and inter-observer variability of the measurement can significantly reduce the manually recorded value's validity. Although the MHC is not a decisive test, the process's simplicity and results determine clinical management, and an incorrect measurement can lead to inappropriate decisions. (10, 11).

Image-based algorithms—such as the one presented here—are currently challenging to implement in hospitals and clinics due to proprietary platforms that rule the transport of medical data. These applications rely on the Picture Archiving and Communications System (PACS) (12). The platform efficiently delivers images to authenticated users in a secure environment that complies with the Health Insurance Portability and Accountability Act (HIPAA) (13). The PACS is currently the standard platform to manage medical images but lacks analytical and quantification capabilities. We have developed an automatic method to retrieve medical data within the PACS and access it at a voxel level (14). This study describes a method to accurately and automatically determine the MHC from MRI studies within the PACS and add that numerical value to the final radiology report as a standard application. An accurate, automatic determination of the MHC from MRI images is of clinical usefulness.

## 2. Materials and Methods

### 2.1. Characterizing the Lack of Reproducibility in MHC Manual Estimation

**Figure 1:**
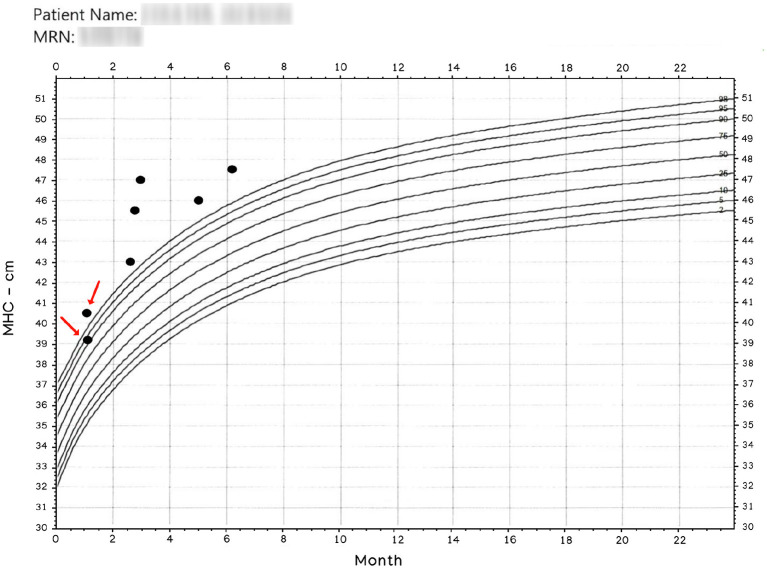
Manually obtained MHC. The x-axis represents age. The entries pointed by arrows were read the same day. Observe that operators have more than 1 cm discrepancy in their readings; nonetheless, a patient classified in and out of normal boundaries is more disturbing. Patient name and MRN have been intentionally blurred.

There is an intuitive certitude that human operators will never yield exact results in manual measurements. Figure 1 was extracted from a medical record, and we employ it here to show the motivation to create the presented method.

Since the results in Figure 1 is anecdotal, we designed a protocol to determine MHC differences in a clinical setting. We asked three trained experts to register their MHC readings from pediatric subjects seen in a neurosurgical clinic. We also kept environmental conditioning during the data collection and noted patients' cooperativeness during the procedure. Each of the three experts independently measured each child's MHC multiple times until it was felt that the most accurate measurement had been obtained. The data presented shows the degree of variance among the three clinicians.

Records of three measurements per subject were gathered for a sample size of *n* = 52. Evalu@ (15) was employed in the data collection tasks to generate real-time statistics and instant remote monitoring. The three experts yielded normal distributions for their manual MHC estimations on 27 males, 25 females within the age range [8–216] months old. We tested the distributions for normality by analytical means using kurtosis, skewness, and the Shapiro-Wilk test. We also verified statistical equals of variance (Leneve test), thus fulfilling ANOVA implementation requirements. Finally, testing statistical differences in the MHC among operators was calculated for a significance α = 0.05.

### 2.2. Phantoms and Synthetic Data Acquisition

**Figure 2:**
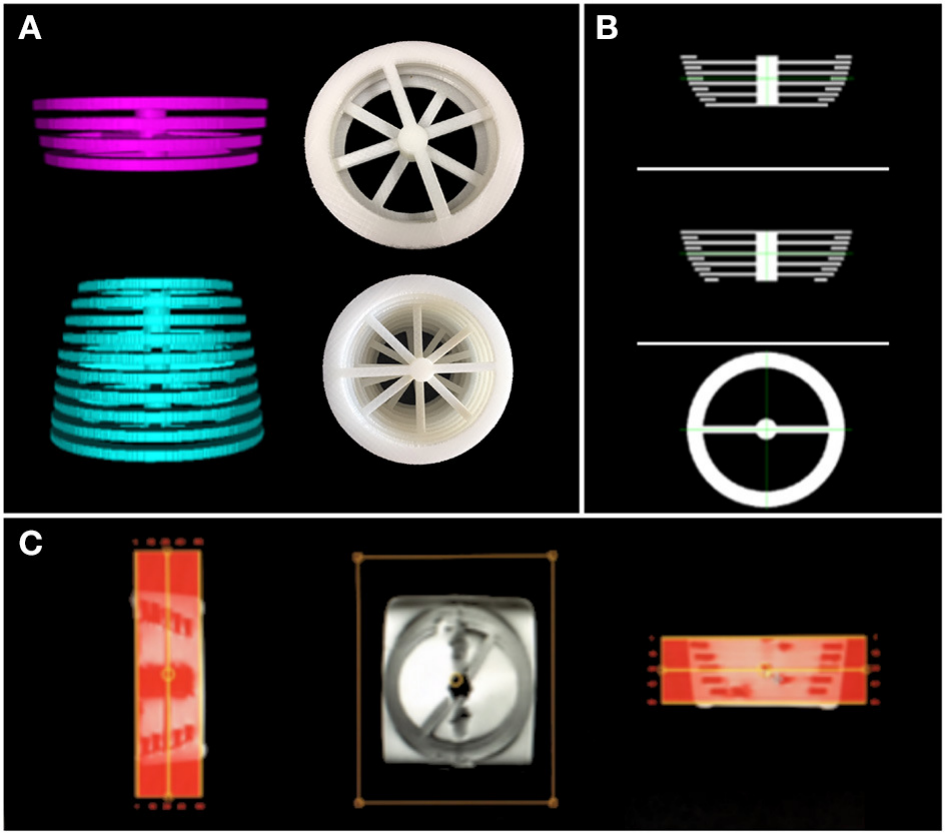
3D structures used to build the MRI phantoms. In **(A)**-top, rings representing MHC from 3 to 18 years old; **(A)**-bottom: rings representing MHC from 0 to 3 years old. In **(A)**, digital prototypes are shown on the left, while 3D-printed creations are presented in the right. **(B)** shows the digital designs of the rings in 3 views. **(C)** is a screenshot of the created MRI phantom obtained from the scanner interface (low resolution). These structures are used to estimate the error induced by the automatic measuring instrument.

We created two 3D structures of 10 and 4 rings (see Figure 2). Both structures were built with rings of 5 mm thickness, and each ring had a different circumference. The perimeters in the structure with ten rings encompass the typical mean head-circumference values registered in the Nellhaus plot for ages ranging from zero to three. The second structure with four rings embraces the Nellhaus circumferences from 3 to 18 years old. The physical structures were created in Blender® with rulers set in 100 μm, saved in StereoLithography (STL) format, converted to Gcode using CURA, and 3D printed with a resolution of 100 μm. Next, the 3D models were used to create MRI phantoms. These MRI phantoms were scanned using typical clinical values for in-plane resolution (0.5 *x* 0.5 *mm*). In turn, the Z-axis was scanned at 1*mm* without spacing, so five slices per ring were obtained. The rings' slices followed the same pipeline presented in this manuscript to obtain the patients-skulls' circumferences. Analytical values of circumference secondary to measuring diameter with a caliper (*precision* = 0.1 *mm*) in the 3D objects and the measurements obtained in the phantom's images are compared to extract the systematic errors induced by the image-based-measuring instrument. A reference to the geometrical technique used to measure the circumference is presented in the discussion to justify the use of perfectly circular shapes in the characterization of non-perfectly circular shapes, as seen in the human's cross-sections skull.

### 2.3. Clinical Data Acquisitions

Magnetic resonance T1-weighted images of 49 children underwent the methods that involve the automatic tool to estimate the MHC. Trained technicians acquired the images in a Philips Achieva 3T scanner; gradient recalled sequence with mag prepared variant. The image parameters, such as voxel resolution, reconstruction matrix, repetition time, echo time, and averages used, are not uniform among the studied sample. We provide a record of voxel dimensions' variability in **Table 4**.

### 2.4. Image Processing

**Figure 3:**
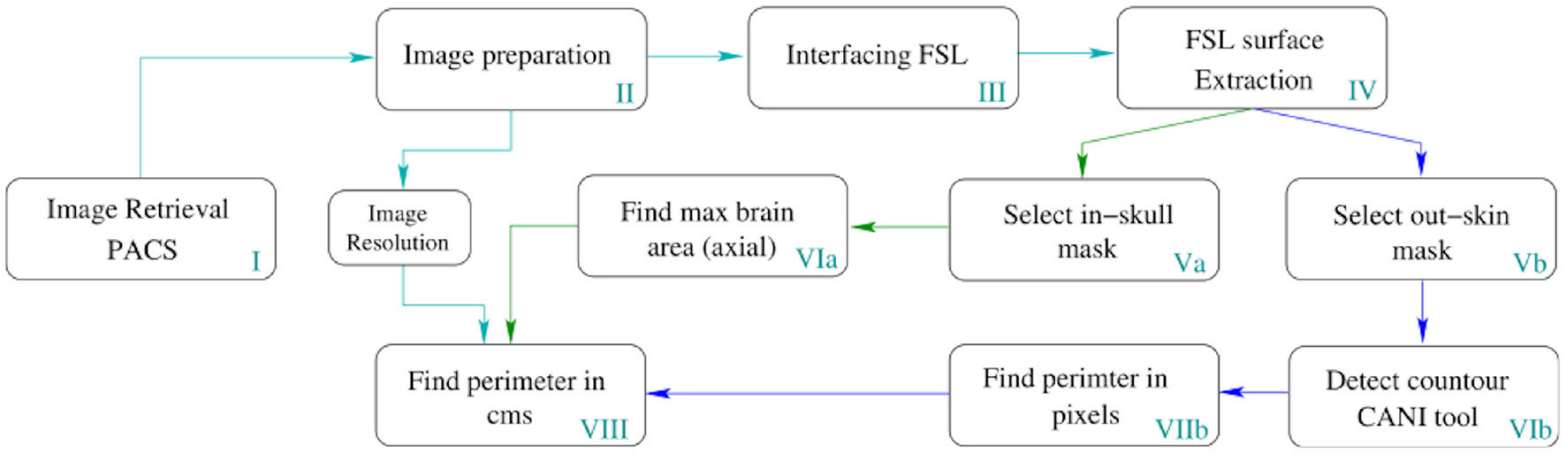
Pipeline for the automatic MHC extraction. The depicted process runs in all slices of the volume but only returns the MHC to place it in the Nellhaus charts. However, this method can measure the perimeter of all the slices comprising the head, enabling the analysis of the whole skull shape to create other indexes.

Image processing was employed to automatically determine the MHC from medical images within the clinical repository. The block diagram in Figure 3, presents a detailed description of the automation.

In Table 1, a step-by-step explanation of the method is provided to assert reproducibility. Block number in Figure 3 and the column step in Table 1 can be associated back and forward. A video running the presented method in a python notebook is provided as Supplementary Material.

**Table 1:** Description of the block diagram shown in Figure 3.

**Step**	**Description**
I	Images are moved after anonymization using the development presented in (14).
II	Image reshape and linear transformation to scanner coordinates is accomplished with Python - nibabel package (16).
III	Python is linked to the Functional Magnetic Resonance Imaging of the Brain Software Library (FSL) (17) using the nipype package (18).
IV	With FSL's functionality, surface extraction is asserted through the BET command.
Va	Recover brain mask.
Vb	Recover out-skin mask.
VIa	Calculate the maximum area in the axial sections found in the whole volume. The max area unit remains in pixels.
VIIb	The perimeter was derived from an ultra-fast hardware implementation in “Design and FPGA implementation of a perimeter estimator” (7).
VIII	In step 1, the original voxel sizes are saved until this point is reached. The perimeter estimation is voxels is transferred to millimeters using the image resolution. The maximum area found in step VIa is used to pick the slice with the MHC.

The technical details discussing the MHC automatic algorithm's interaction and how it is connected to the PACS vehicle to create a holistic solution in the highly regulated medical environment are provided in (14).

### 2.5. Manual vs. Automatic Assessments of MHC

The study was executed at *Children Hospital Los Angeles* (IRB #: CHLA-15-00161). It involved a retrospective chart review of the neurosurgical database; therefore, informed consents were unnecessary. However, all medical information was previously anonymized. After filtering with terms related to hydrocephalus or aberrant CSF circulation, patients (*n* = 49) were selected from the internal neurosurgical database. Only patients in whom a manual MHC measurement and an MRI study were performed on the same day are part of this study. This timing controlled for possible discrepancies caused by head growth in the youngest subjects. The manually performed records of MHC were determined and compared with those obtained automatically from MRI scans by the device presented in this paper. We provide statistical analysis to compare manual and automatic results. Data gathering, comparisons and device adjustments were executed with Python® using numpy® (19), scipy® (20), and pandas® (21) packages.

## 3. Results

### 3.1. Errors in MHC Manual Estimation

In this section, we gather manual MHC records and study their distributions to determine statistical differences among operators. Table 2 resumes the analysis on the measurements of MHC performed by three experts.

**Table 2:** Statistical analysis on samples of MHC obtained manually by three experts.

**Sample origin**	**Test**	**Hypothesis or decision criteria**	**Obtained values**	**Interpretation**
GM MHC	Kurtosis and skewness	In range [−1,1]	[−0.18,0.44]	Within the range; thus distributions are normal (22)
JK MHC			[−0.50,0.26]	
AS MHC			[−0.19,0.37]	
GM MHC	Shapiro–Wilk	*H*_0_: Data is normal α = 0.05 Discard *H*_0_ if *p* ≤ α	*p* = 0.280	Assume normal distributions
JK MHC			*p* = 0.384	
AS MHC			*p* = 0.411	
All origins	Leneve	*H*_0_: Data has equal variance α = 0.05 discard *H*_0_ if *p* ≤ α	*p* = 0.957	Assume equal variance among all groups
All origins	ANOVA	*H*_0_:*u*1 = *u*2 = *u*3 α = 0.05 Discard *H*_0_ if *p* ≤ α	*f* = 3.17 *p* = 0.044	Samples of MHC are statistically different

*Sample size n = 52. GM, JK, and AS are the initials of the experts*.

In Table 2, the ANOVA test discarded the null hypothesis (*p* = 0.044, α = 0.05), consequently the prospective measurements taken by the experts in the randomly selected cohort of 52^1^ subjects, yielded statistically different results.

### 3.2. Tool Calibration

The rings' circumferences of the phantoms depicted in Figure 2 were measured using the method described in this manuscript. Each ring generated five readings without the need for re-sampling; therefore, the instrument's precision was determined by the variability among five readings per ring. See the column for estimated value (Est. Value) in Table 3. The accuracy of the measuring method was calculated by comparing the estimated value with the physical value. See the column Real Value after measuring diameter *D* with a caliper and using *C* = *Dπ* in Table 3. The difference column is employed to compute a correction factor (CF). One would expect more substantial errors in bigger circumferences, but this was not the case within-group estimations among the two testing structures—0 to 3 and 3 to 18 years old. However, there are inter-group differences which motivated the use of two correction factors (CFs). These CF values are presented as a mean and standard deviation pair obtained by computing the difference values in each group. The obtained CFs were applied depending on the age of the patient. The correction mechanics and effects on the measurement can be appreciated in Table 3.

**Table 3:** Record of the differences encountered between the automatically obtained circumferences (column Est. Value) and the physical circumferences of the rings in the phantoms (column Real Value).

**Item**	**Structure 1 (9 rings, 0–3 years)**							
	**Read 1**	**Read 2**	**Read 3**	**Read 4**	**Read 5**	**Est. value**	**Real value**	**Difference**
Ring 1	37.88	38.47	37.91	38.14	38.06	38.1 ± 0.2	35.5 ± 0.3	-2.6 ± 0.4
Ring 2	41.96	41.27	42.04	41.75	41.15	41.6 ± 0.4	39.3 ± 0.3	-2.4 ± 0.5
Ring 3	44.55	44.40	44.75	44.33	44.72	44.6 ± 0.2	41.8 ± 0.3	-2.8 ± 0.4
Ring 4	46.68	46.29	46.06	46.34	45.82	46.2 ± 0.3	43.4 ± 0.3	-2.9 ± 0.4
Ring 5	47.71	48.17	48.35	47.89	48.16	48.1 ± 0.3	45.6 ± 0.3	-2.4 ± 0.4
Ring 6	48.32	48.83	49.24	48.96	49.62	49.0 ± 0.5	46.5 ± 0.3	-2.5 ± 0.6
Ring 7	50.55	50.18	50.35	50.64	49.41	50.2 ± 0.5	47.7 ± 0.3	-2.5 ± 0.6
Ring 8	50.51	51.48	50.51	50.65	51.07	50.9 ± 0.4	48.4 ± 0.3	-2.5 ± 0.5
Ring 9	52.71	52.08	51.89	51.72	52.12	52.1 ± 0.4	49.3 ± 0.3	-2.8 ± 0.5
**Item**	**Structure 2 (4 rings, 3–18 years)**							
	**Read 1**	**Read 2**	**Read 3**	**Read 4**	**Read 4**	**Est. value**	**Real value**	**Difference**
Ring 10	55.28	54.46	54.67	54.34	54.08	54.6 ± 0.5	51.2 ± 0.3	-3.2 ± 0.6
Ring 11	56.36	56.58	55.69	56.58	56.70	56.4 ± 0.4	52.8 ± 0.3	-3.6 ± 0.5
Ring 12	58.11	58.52	58.11	58.22	58.24	58.2 ± 0.2	54.7 ± 0.3	-3.6 ± 0.4
Ring 13	59.48	58.67	58.34	58.52	59.47	58.9 ± 0.5	55.3 ± 0.3	-3.6 ± 0.6

*All values are in centimeters (cm). The label Est. Value stands for Estimated Value*.

### 3.3. Clinical Measurement

Single results of measuring the MHC in the clinical data for the randomly selected subjects were registered in Table 4. All records are in centimeters (cm). The average difference between automatic and manually read perimeters in girls (*n* = 20) was 1.7 ± 0.12, with a maximum of 3.0 ± 0.52. The same comparison was performed in boys' perimeters (*n* = 29) yielding an average difference of 1.6 ± 0.09, with a maximum of 2.6 ± 0.52. The difference between manual and MRI automatically estimated data (*n* = 49) was 1.6 ± 0.07, with a maximum equal to 3.0 ± 0.52, all units in centimeters (cm).

**Table 4:** Comparison between manually and automatically obtained MHCs using correction factors.

**S**	**Res**.	**Pos**.	**MHCE**		**Diff1**	**CF**	**New HC**	**Diff2**
			**Manual**	**Automatic**				
1	0.58 × 0.58 × 5.00	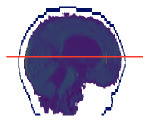	57.1 ± 0.1	61.3 ± 0.5	−4.2 ± 0.5	−3.7 ± 0.3	57.6 ± 0.6	−0.5 ± 0.6
2	0.41 × 0.41 × 4.00	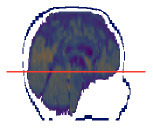	44.8 ± 0.1	46.7 ± 0.5	−1.9 ± 0.5	−2.8 ± 0.2	43.9 ± 0.5	0.9 ± 0.5
3	0.58 × 0.58 × 5.00	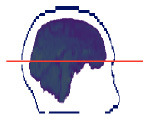	49.0 ± 0.1	53.3 ± 0.5	−4.3 ± 0.5	−3.7 ± 0.3	49.6 ± 0.6	−0.6 ± 0.6
4	0.85 × 0.85 × 3.99	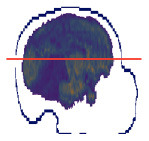	48.5 ± 0.1	52.7 ± 0.5	−4.2 ± 0.5	−3.7 ± 0.3	49.0 ± 0.6	−0.5 ± 0.6
5	0.46 × 0.46 × 4.99	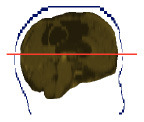	57.1 ± 0.1	60.1 ± 0.5	−3.0 ± 0.5	−3.7 ± 0.3	56.4 ± 0.6	0.7 ± 0.6
6	0.57 × 0.57 × 4.99	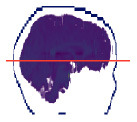	54.6 ± 0.1	57.6 ± 0.5	−3.0 ± 0.5	−3.7 ± 0.3	53.9 ± 0.6	0.7 ± 0.6
7	0.79 × 0.79 × 4.99	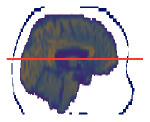	56.0 ± 0.1	59.1 ± 0.5	−3.1 ± 0.5	−3.7 ± 0.3	55.5 ± 0.6	0.5 ± 0.6

In Table 4 the labels S, MHCE, Diff, CF, and HC, stand for: subject, maximum head circumference estimation, difference, correction factor, and head circumference, respectively. All values are in centimeters (cm). The seven subjects shown were selected among the 49 samples due to their remarked abnormal shapes (not necessarily out of the normal circumference range). Abnormality of this sort is more challenging for automation; however, the proposed method demonstrated robustness against the most intricate shapes found in this clinical sample.

### 3.4. Statistical Analysis on Manual and Automatic MHC Measurements

In like manner to section 3.1, we analyzed the gathered data to derive a concept of group equality at a 95% confidence level. Table 5 shows the results of the statistical exercise.

**Table 5:** Comparing the MHC measurement perform by human operators and the automatic estimator for a sample size *n* = 49.

**Sample origin**	**Test**	**Hypothesis or decision criteria**	**Obtained values**	**Interpretation**
Manual MHC	Kurtosis and skewness	In range [−1,1]	[−0.25,0.01]	Within the range; Samples are normal
Automatic MHC			[−0.11,0.16]	
Manual MHC	Shapiro-Wilk	*H*_0_: Data is normal α = 0.05, discard *H*_0_ if *p* ≤ α	*p* = 0.554	Assume normal distributions
Automatic MHC			*p* = 0.767	
All origins	Leneve	*H*_0_: data has equal variance α = 0.05 discard *H*_0_ if *p* ≤ α	*p* = 0.654	Assume equal variance among all groups
All origins	*T*-test	*H*0:*u*1 = *u*2 α = 0.05 discard *H*_0_ if *p* ≤ α	*t*_*score*_ = 0.924 *p* = 0.357	Can not discard *H*_0_

In Table 5, the *T*-test could not discard the null hypothesis (*p* = 0.357, α = 0.05); consequently, there is not statistical difference between the manual and the automatic estimation of MHC in the randomly selected cohort.

### 3.5. Automatically Registering MHC in Radiology Reports and the Patient's Medical Record

**Figure 4:**
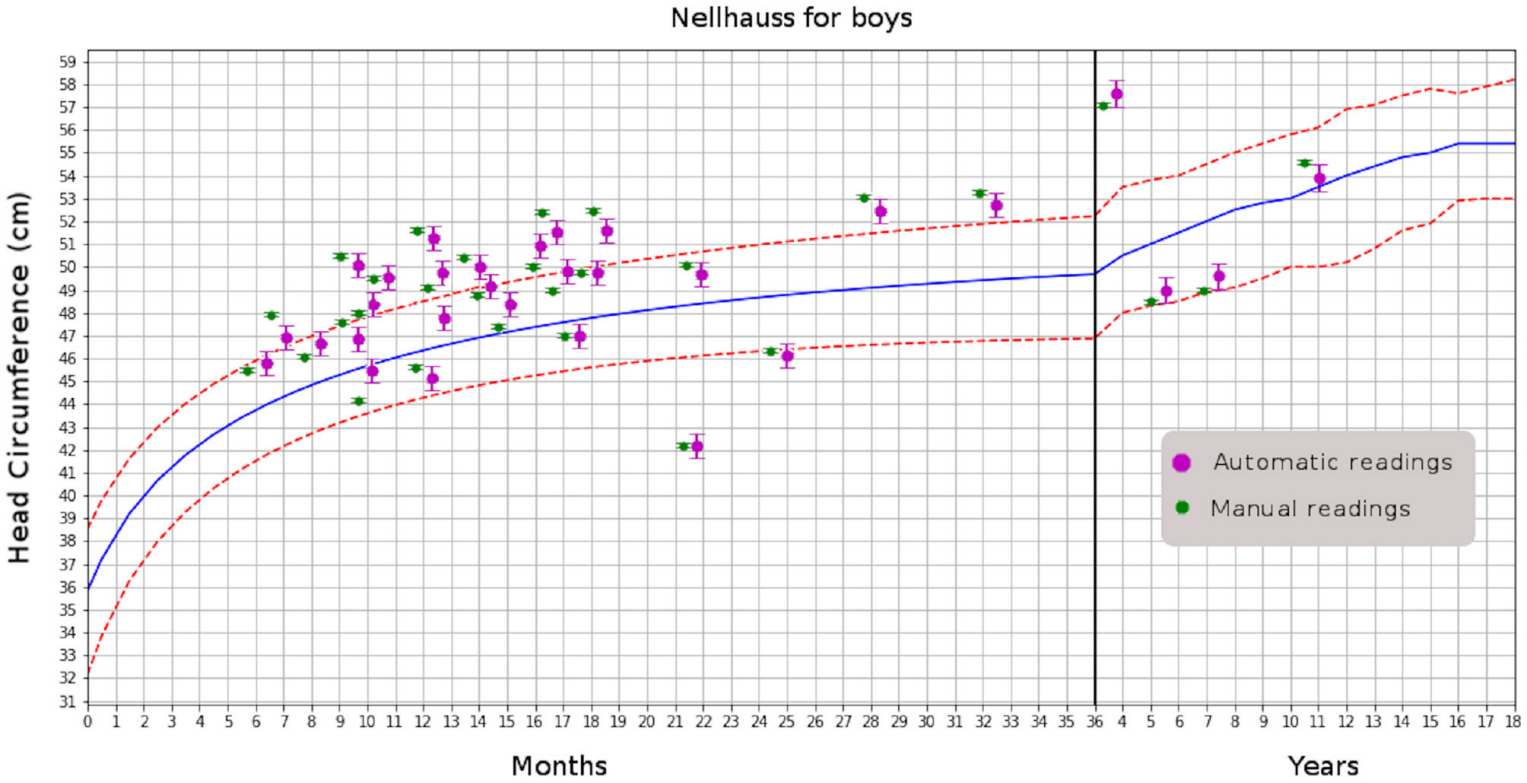
Comparison of automatic and manual readings for boys. That for girls has similar distributions.

After achieving accuracy in MHC determination from MRI studies, the next step is to automatically include the radiology report's measurement and plot it on a head-circumference chart. Manually generated MHC measurements can be added to the medical report as well. It will also be possible to compare the current patient with others in the same age range or suffering from the same abnormality; for instance, examining patients with various forms of craniosynostosis to evaluate head growth in response to different surgical correction methods. In the automation, we also included plotting using a digitally created version of the Nellhaus chart (see Figure 4)

## 4. Discussion

MR images' quality is compromised by acquisition time; however, minimizing scan time is essential when working with neonates, infants, children, and uncooperative non-sedated patients. Additionally, ultra-fast multi-echo sequences reduce the artifacts created by the patient's motion and generate structural images of acceptable quality in less than 3 min (23), favoring the feasibility of the methods presented in this document.

There are concerns regarding the use of automatic measurements since the clinical images have a relatively low resolution. However, for the MHC calculations, the images have an acceptable in-plane resolution around half of a millimeter with isometric pixels. The low resolution is a mechanism to accelerate the acquisitions and happens in the z-axis, where the spacing of 4–5 mm is standard. Z-axis defines slice thickness and may mislead the location of the MHC in axial cuts. Such a source of error introduces a *slice* − *thickness*/2 mm factor of localization uncertainty. Since skull-shape modifiers do not happen abruptly within the *slice* − *thickness*/2 mm of error—considering operational values of slice-thickness—the proposed method will always be capable of locating the MHC. Parallax errors are avoided by spatial image standardization.

Another possible drawback has to do with the use of perfectly rounded shapes during the instrument-tuning activities. Arguably, an ellipse is a better representation of the typical head shape. However, the errors attained by digitally measuring a rounded boundary are the same whether the boundary belongs to a circle or an ellipse (24). Calculation of the perimeters of ellipse-like shapes is challenging and presents another complication for their use in this analysis. While the circles' geometry has deterministic theoretical values, the formulation in the ellipse is not deterministic and always carries an approximation (25).

It is worth mentioning that once the instrument is tuned –using the 3D models—additional tuning sessions are not necessary to measure MHC on new patients. Hardware assisting instruments p.e, voltmeters, MRI bores, and power backups, among several others, are certified in their range of operation by the maker and might need adjustment every year due to the natural wear of components. Instead, software instruments like the one proposed, once delivered, remain operational with factory specifications forever.

The gold standard and phantoms creation is a solid strategy to certify the instrument's accuracy and one can use it when reproducible devices lack accuracy.

The Nellhaus curves were proposed in 1968 and have been commonly used in pediatric units ever since. Other studies reproducing the Nellhaus chart have been presented from 1987 to 2000 (26, 27), including digital files provided by the Centers for Disease Control and Prevention (CDC) (28). Nonetheless, medical personnel still perform the MHC manually even at hospitals with excellent technological setups.

More recently, automatic methods with sophisticated approaches have been proposed in (29–31). These authors include powerful strategies for skull boundary detection, such as random forest and regressions to predict the perimeters. Some others worked on the stable CT Hounsfield units that facilitate the detection of boundaries by thresholding, a technique with few or no applicability in other image diagnosing modalities of extensive use due to variable spin recruitment in MRI and operator-dependent echos in ultrasound.

In general, the solutions found in the literature might be sufficient for the task, but since they are validated with manual segmentation, we do not know how accurate they are. In contrast, our tuning strategy using 3D physical models displays a certifying framework where the instruments are tested for accuracy in the measurement range.

Another essential aspect when creating technological tools has to do with the development platform. The cited authors created their solutions using Matlab®, an outstanding tool for prototyping, but impedes rapid transferring to production stages. We instead used Python® in this automation, since clinical usability was within the project's objectives.

PACS policies are crucial for implementation of automatic solutions. The cited proposals do not consider the regulations regarding confidentiality. In medical networks. Our implementation is CAPS-compatible via the method introduced in (14), as physical layer connectivity is exploited; thus, PACS vendor independence is achieved.

The current work is not intended to find if a variable resides within the acceptable boundaries of a statistical range defined by human measurements. Such a scheme is useless because humans would yield fluctuating values even if the same operator performs the task multiple times. In turn, this work presents an automatic device's design and a strategy that uses objects with known dimensions to tune the measuring instrument.

Note in Table 5 that manual and automation measured with same statistical accuracy. This is a 95% confidence test executed over a very limited sub-sample of the data. There is still a 5% of statistical probability that patients receive a manual MHC differing with the automation in a value that moves the reading beyond the line of changing a verdict. With the tuned device, one can declare a correct value, something unfeasible with manual assessments.

## 5. Conclusions

Manually obtained head circumference measurements are operator-dependent in all cases. As our approach is fully automatic, it assures reproducibility and accuracy after tuning. Moreover, the presented implementation is conducted with full PACS compatibility, and the results can be automatically included in the radiology report. Also, due to the PACS compatibility, the MHC value's usability can be extended to monitor a given patient over time or compare it with other patients in a given database. The presented method does not advocate for the use of MRI or CT to obtain the MHC; instead, it uses images saved in the hospital database acquired for clinically indicated purposes. The validation process involves a strategy that can be interpolated to certify the accuracy of devices extracting geometries in medicine and other fields using imaging.

## Data Availability Statement

The raw data supporting the conclusions of this article will be made available by the authors, without undue reservation.

## Ethics Statement

The studies involving human participants were reviewed and approved by IRB: CHLA-15-00161. Written informed consent for participation was not required for this study in accordance with the national legislation and the institutional requirements.

## Author Contributions

FY-C: hypothesis, conception, experiment design, programming, data processing, and writing. FW: data gathering and data processing. AS: data gathering and manual MHC testing. JK: data gathering, data verification, and data processing. MN: hypothesis, conception, and administration. JGM: hypothesis, conception, administration, document revision, writing, and data gathering. All authors contributed to the article and approved the submitted version.

## Conflict of Interest

Strategic Business Platforms LLC employs FY-C since September 2019 after five years of research at the Neurosurgery Division of the Children's Hospitals Los Angeles. The remaining authors declare that the research was conducted in the absence of any commercial or financial relationships that could be construed as a potential conflict of interest.

## Footnotes

^1^Two groups of images were gathered in this research. This addition avoids confusing the readers with the sample numbers.

## References

[1] de Onis M. WHO Child Growth Standards. Methods and Development. World Health Organization (2009). Available online at: http://www.who.int/childgrowth/standards/velocity/tr3_velocity_report.pdf?ua=1

[2] Menounou A. Head size: is it important? Adv Clin Neurosci Rehabil. (2011) 2:16–20.

[3] Illingworth RS Lutz W. Head circumference of infants related to body weight. Arch Dis Child. (1965) 40:672–6. 10.1136/adc.40.214.672 5844957 PMC2019471

[4] Poca MA Martínez-Ricarte FR Portabella M Torné R Fuertes ML González-Tartiere P. Head circumference: the forgotten tool for hydrocephalus management. A reference interval study in the Spanish population. Clin Neurol Neurosurg. (2013) 115:2382–7. 10.1016/j.clineuro.2013.09.001 24070639

[5] Kariminejad A Kariminejad R Tzschach A Ullmann R Ahmed A Asghari-Roodsari A. Craniosynostosis in a patient with 2q37.3 deletion 5q34 duplication: association of extra copy of MSX2 with craniosynostosis. Am J Med Genet. (2009) 149A:1544–49. 10.1002/ajmg.a.32949 19533795

[6] Coronado R Macaya A Giraldo J Roig-Quilis M. Concordance between a head circumference growth function and intellectual disability in relation with the cause of microcephaly. An Pediatr. (2015) 83:109–16. 10.1016/j.anpedi.2014.10.030 25534043

[7] Leibovitz Z Spiegel ED Malinger G Haratz K Tamarkin M Gindes L. Prediction of microcephaly at birth using three reference ranges for fetal head circumference: can we improve prenatal diagnosis? Ultrasound Obstret Gynecol. (2015). 47:586–92. 10.1002/uog.15801 26511765

[8] Sacco R Stefano Gabriele Persico AM. Head circumference and brain size in autism spectrum disorder: a systematic review and meta-analysis. Psychiatry Res Neuroimaging. (2015) 234:239–51. 10.1016/j.pscychresns.2015.08.016 26456415

[9] Samuelsen SO Bakketeig LS Tretli S Johannesen TB Magnus P. Head circumference at birth and risk of brain cancer in and childhood: a population-based study. Lancet Oncol. (2006) 7:39–42. 10.1016/S1470-2045(05)70470-8 16389182

[10] Hofmann B Welch HG. New diagnostic tests: more harm than good. BMJ. (2017) 358:j3314. 10.1136/bmj.j3314 28720607

[11] Sachdeva RC Jain S. Making the case to improve quality and reduce costs in pediatric health care. Pediatr Clin N Am. (2009) 56:731–43. 10.1016/j.pcl.2009.05.013 19660624

[12] Yepes-Calderon F Wihardja F Melamed E Song M Paladini G Lepore N. Extending PACS functionality: towards facilitating the conversion of clinical necessities into research-derived applications. In: Proc. SPIE. (2017) p. 1016015–8. 10.1117/12.2264350 31178617 PMC6554204

[13] Rachel Nosowsky Giordano TJ. The Health Insurance Portability and Accountability Act of (1996). (HIPAA) privacy rule: implications for clinical research. Annu Rev Med. (2006). 57:575–90. 10.1146/annurev.med.57.121304.131257 16409167

[14] Yepes-Calderon F Bluml S Erberich S Nelson MD McComb JG. Improving the picture archiving and communication system: towards one-click clinical quantifying applications. Comput Methods Biomech Biomed Eng Imaging Visual. (2018) 7:154–61. 10.1080/21681163.2018.1466199

[15] Yepes-Calderon F Zuluaga JFY Calderon GEY. Applied informatics ICAI (2019). In: Florez H Leon M Diaz-Nafria JM Belli S, editors. Evalu@: An Agnostic Web-Based Tool for Consistent and Constant Evaluation Used as a Data Gatherer for Artificial Intelligence Implementations. Madrid: Springer (2019). 10.1007/978-3-030-32475-9_6

[16] Van Rossum G Drake FL. Python 3 Reference Manual. Scotts Valley, CA: CreateSpace (2009).

[17] Jenkinson M Beckmann CF Behrens TEJ Woolrich MW Smith SM. FSL. Neuroimage. (2012) 62:782–90. 10.1016/j.neuroimage.2011.09.015 21979382

[18] Krzysztof G Christopher B Cindee M Dav C Yaroslav H Michael W. Nipype: a flexible, lightweight and extensible neuroimaging data processing framework in python. Front Neuroinform. (2011) 5:13. 10.3389/fninf.2011.00013 21897815 PMC3159964

[19] Harris CR Millman KJ van der Walt SJ Gommers R Virtanen P Cournapeau D. Array programming with NumPy. Nature. (2020) 585:357–62. 10.1038/s41586-020-2649-2 32939066 PMC7759461

[20] Virtanen P Gommers R Oliphant TE Haberland M Reddy T Cournapeau D. SciPy 1.0: fundamental algorithms for scientific computing in Python. Nat Methods. (2020) 17:352. 10.1038/s41592-020-0772-5 32094914 PMC7056641

[21] McKinney W. Data structures for statistical computing in Python. In: van der Walt S Millman J, editors. Proceedings of the 9th Python in Science Conference. Austin, TX (2010). p. 51–6. 10.25080/Majora-92bf1922-00a

[22] Bulmer MG. Principles of Statistics. Dover Books on Mathematics. Dover Publications (2012). Available online at: https://books.google.com.co/books?id=BZi8AQAAQBAJ

[23] Pai VM. Multi-Echo Magnetic Resonance Imaging Method and System. Google Patents (2011). US Patent US8060180B2. Available online at: https://patents.google.com/patent/US8060180B2/en

[24] Benkrid K Crookes D Benkrid A. Design and FPGA implementation of a perimeter estimator. In: *Proceedings of the Irish Machine Vision and Image Processing Conference*. IT Sligo (2000). p. 51–7.

[25] Wang MK. Precise estimates for the solution of Ramanujan's generalized modular equation. Ramanujan J. (2019) 49:653–68. 10.1007/s11139-018-0130-8

[26] Roche AF Mukherjee D Guo S Moore WM. Head circumference reference data: birth to 18 years. Pediatrics. (1987) 79:706–12. 3575026

[27] Farkas LG Posnick JC Hreczko TM. Anthropometric growth study of the head. Craniofac J. (1992). 29:208–315. 10.1597/1545-1569_1992_029_0303_agsoth_2.3.co_2 1643057

[28] Flegal KM Cole TJ. Construction of LMS parameters for the Centers for Disease Control and Prevention 2000 growth charts. Natl Health Stat Rep. (2013) 63:1–3. 24992748

[29] Li J Wang Y Lei B Cheng ZJ Qin J Wang T. Automatic fetal head circumference measurement in ultrasound using random forest and fast ellipse fitting. IEEE J Biomed Health Inform. (2017) 22:215–23. 10.1109/JBHI.2017.2703890 28504954

[30] Smith K Politte D. Automated measurement of skull circumference, cranial index, and braincase volume from pediatric computed tomography. In: 35th Annual International Conference of the IEEE EMBS. Osaka (2013). p. 3–7. 10.1109/EMBC.2013.6610416 PMC4559142 24110603

[31] Vorperian HK Durtschi RB Wang S Chung MK Ziegert AJ Gentry LR. Estimating head circumference from pediatric imaging studies: an improved method. Acad Radiol. (2007) 14:1102–7. 10.1016/j.acra.2007.05.012 17707318

